# Prevalence and risk factors for *Giardia duodenalis *infection among children: A case study in Portugal

**DOI:** 10.1186/1756-3305-5-22

**Published:** 2012-01-27

**Authors:** Cláudia Júlio, Anabela Vilares, Mónica Oleastro, Idalina Ferreira, Salomé Gomes, Lurdes Monteiro, Baltazar Nunes, Rogério Tenreiro, Helena Ângelo

**Affiliations:** 1 National Institute of Health Dr. Ricardo Jorge, Department of Infectious Diseases, National Reference Laboratory for Gastrointestinal Infections, Av. Padre Cruz, Lisbon, Portugal; 2 National Institute of Health Dr. Ricardo Jorge, Department of Infectious Diseases, Research and Development Unit, Av. Padre Cruz, Lisbon, Portugal; 3 National Institute of Health Dr. Ricardo Jorge, Department of Epidemiology, Av. Padre Cruz, Lisbon, Portugal; 4 University of Lisbon, Faculty of Sciences, BioFIG, Edifício ICAT, Campus da FCUL, Campo Grande, Lisbon, Portugal

**Keywords:** *Giardia duodenalis*, prevalence, risk factors, children, Portugal

## Abstract

**Background:**

*Giardia duodenalis *is a widespread parasite of mammalian species, including humans. The prevalence of this parasite in children residing in Portugal is currently unknown. This study intended to estimate *G. duodenalis *infection prevalence and identify possible associated risk factors in a healthy paediatric population living in the District of the Portuguese capital, Lisbon.

**Methods:**

Between February 2002 and October 2008, 844 children were randomly selected at healthcare centres while attending the national vaccination program. A stool sample and a questionnaire with socio-demographic data were collected from each child. *Giardia *infection was diagnosed by direct examination of stools and antigen detection by ELISA.

**Results:**

The population studied revealed a gender distribution of 52.8% male and 47.2% female. Age distribution was 47.4% between 0-5 years and 52.6% between 6-15 years.

The prevalence of *Giardia *infection was 1.9% (16/844) when estimated by direct examination and increased to 6.8% (57/844) when ELISA results were added. The prevalence was higher among children aged 0-5 years (7.8%), than among older children (5.8%), and was similar among genders (6.9% in boys and 6.5% in girls). The following population-variables were shown to be associated risk factors for *G. duodenalis *infection: mother's educational level (odds ratio (OR)= 4.49; confidence interval (CI): 1.20-16.84), father's educational level (OR = 12.26; CI: 4.08-36.82), presence of *Helicobacter pylori *infection (OR = 1.82; CI: 1.05-3.15), living in houses with own drainage system (OR = 0.10; CI: 0.02-0.64) and reported household pet contact, especially with dogs (OR = 0.53; CI: 0.31-0.93).

**Conclusion:**

The prevalence of giardiasis in asymptomatic children residing in the region of Lisbon is high. Several risk factors were associated with *Giardia *prevalence and highlight the importance of parents' education and sanitation conditions in the children's well being. The association between *G. duodenalis *and *H. pylori *seems an important issue deserving further investigation in order to promote prevention or treatment strategies.

## Background

The intestinal protozoan *Giardia duodenalis *(synonym of *Giardia intestinalis *and *Giardia lamblia*) is a cosmopolitan parasite frequently found in diarrhoeal disease throughout the world [1]. It is one of the most common causes of waterborne disease outbreaks associated with drinking water [2,3]. The prevalence of giardiasis is 2 to 5% in developed countries and 20 to 30% in developing countries [4]. The high prevalence in these countries has been suggested to be associated with long-term growth retardation in children [5]. In the United States of America and United Kingdom, *G. duodenalis *is the most commonly reported intestinal protozoan in humans [6], mainly affecting children. The pathogenesis is not clearly understood and symptoms, including acute or chronic diarrhoea, dehydration or abdominal pain, are highly variable [3,7], and may even not be evident in a significant proportion of infected patients [8]. In children less than five years old, *Giardia *infection may produce severe acute diarrhoea. On the other hand, chronic infection may result in weight loss and growth retardation [9].

The cysts are the environmentally stable stage and are resistant to inactivation by drinking water disinfectants, remaining viable for up to two months [10]. The relative contribution of person-to-person, animal-to-person, foodborne and waterborne transmission to sporadic human giardiasis remains unclear [3,11].

The diagnosis is initially based on clinical signs and symptoms and confirmed by the presence of cysts and trophozoites in stool samples. There is no gold standard for the diagnosis of giardiasis [12]. Historically, trophozoites or cysts of *G. duodenalis *were detected in stool samples by microscopic examination (direct examination), whereas antigen detection of stools by enzyme immunoassay (ELISA) is a more sensitive method, recently developed [13]. A definitive diagnosis may require repeated stool examinations, stool immunoassays, or even sampling of the upper intestinal contents [14].

Although *G. duodenalis *is recognized as a common parasite in humans in Portugal (Antunes, *personal communications*), the actual prevalence of this infection in children residing in Portugal remains unknown.

The goal of the present work is to estimate the *G. duodenalis *infection prevalence and identify possible associated-risk factors in a healthy paediatric population living in the Lisbon area. This is the first comprehensive study in children residing in Portugal, analysing a group of infants and children attending the national vaccination programme in the District of Lisbon, in order to assess factors predisposing to *Giardia *infection as well as co-infection with potential enhancing microorganisms.

## Methods

### Stool samples

Between February 2002 and October 2008, 844 healthy children were randomly selected at 15 health care centres from the urban and rural areas of the District of Lisbon, while attending the national vaccination program. In order to participate in the study, parents gave written informed consent, following an explanation of the study aims and procedures. For each participating child, one stool specimen was collected. At the time of specimen collection, parents completed a written questionnaire with information regarding demographic data, personal details of housing and living conditions, contact with pets, child and parents' level of education and any recent illness of children was also obtained. A stool sample was collected from each child, transferred into a sterile universal plastic container, and sent to the Infectious Diseases Department of the National Institute of Health (INSA), in Lisbon, within 24 h of specimen collection. Specimens were aliquoted and conserved at -80'C until tested, and the remaining sample was used for direct examination.

### Ethical Approval

This study received the approval of INSA's Ethics Committee.

### Determination of Giardia prevalence

Fresh stool samples were used to prepare a wet mount and analysed for the presence of *G. duodenalis *cysts by direct microscopic examination (direct examination). All detected cysts were identified according to morphological characteristics under light microscopy using 400 × magnification. Stool antigen detection was performed with Ridascreen *Giardia *enzyme immunoassay (ELISA) test (R-Biopharm AG, Landwehrstr, Darmstadt) according to the manufacturer's instructions [15,16]. One positive and two negative controls were used at each run. Optical density (OD) was measured with an automatic microplate spectrophotometer (Spectramax, Molecular Devices, USA). A positive result was defined as an OD reading 10% over the cut-off value (negative control OD + 0.15), according to the manufacturer's instructions.

### Statistical analysis

The variables analysed potentially associated with *G. duodenalis *infection were: gender, age, the number of adults and children in the family, birth order of the child, mother's and father's educational level, attendance of a day care institution/kindergarten, the child medical status, residence (rural *versus *urban), housing, and contact with pets. All statistical analysis was performed with SPSS software for Windows 18.01 (2009). Results were analysed by the Fisher Exact test, and differences between two proportions were compared. A probability under 0.05 was considered significant. The variables were calculated with their adjusted odds ratios (OR), 95% confidence intervals and significance levels. A multiple logistic regression analysis was performed to study the independence of the association of the variables with a significant *p *value. The level of significance of *p *< 0.05 was set for multivariable analysis; the model was interpreted using adjusted ORs and 95% CIs.

## Results

The study included 844 children, comprising 446 (52.8%) boys and 398 (47.2%) girls. Children were stratified in two age groups: 403 (47.7%) children age 0 (1 month) -5 years, 441 (52.3%) children age 6-15 years (Table 1).

**Table 1:** Distribution of children according to gender and age.

Gender	Male	Female	Total
**Age**			
**0 - 5 years**	49.4% (199)	50.6% (204)	47.7% (403)
**6 - 15 years**	56.0% (247)	44.0% (194)	52.3% (441)
**Total**	52.8% (446)	47.2% (398)	100% (844)

A stool sample was considered positive for *G. duodenalis *if at least one test was positive. Results showed that 16 out of 844 (1.9%) stool samples were positive by direct examination. Using the monoclonal ELISA method, 54 out of 807 (6.7%) were positive. The small size of sample in 37 samples prevented the use of the ELISA method. Three samples revealed as positive by microscopy were negative using ELISA; on the other hand 42 samples were negative by microscopy but positive using ELISA. Overall, 57 out of 844 samples (6.8%) were positive for *G. duodenalis *infection. When age and gender were considered, the prevalence was: 7.8% (31/396) among children aged 0-5 years and 5.8% (25/439) in the oldest ones (6-15); 6.9% (31/447) for males and 6.5% (26/397) for females.

Parents' educational level was found to be highly associated with the prevalence of giardiasis among children. Actually, children with mothers without any education were more likely to be infected with *G. duodenalis *than those with educated mothers (*p *= 0.047; OR = 4.49; 95% CI: 1.20-16.84). Similarly, children living with fathers with no education had a 12.26 times higher risk (*p *< 0.001; 95%CI: 4.08-36.82) of being infected with *Giardia *than those with educated fathers (Table 2). On the other hand, children living in houses with their own drainage system were more protected from this infection (*p *= 0.038; OR = 0.10; 95%CI: 0.02-0.64). Children were also tested for *H. pylori *infection by antigen stool detection [17], and co-infection was detected in 25 children (p < 0.037; OR = 1.82; 95%CI: 1.05-3.15). A sub-set of children negative for *H. pylori *infection were monitored every 6 months, during 36 months, in order to evaluate the incidence of *H. pylori *infection [16]. This group of children was also tested for *Giardia *infection, using the same methods described above. Results showed that 11 children were infected with both *Giardia *and *H. pylori*, with two of these cases positive for both agents in the 18-months sample. Also, 8 of those children were first infected with *Giardia *and then with *H. pylori*, with a time-frame of 6 months (n = 5), 18 months (n = 1) and 30 months (n = 2); one child was first infected with *H. pylori *and then with *Giardia *6 months later.

**Table 2:** Prevalence of *Giardia duodenalis *infection according to demographic data and family characteristics.

Risk factor (total n*)	n	Positive results (%)	*p*	Risk Estimate (OR)	OR Confidence Interval (95%)
**Gender (844)**					
Male	446	31 (6.9)	0.891	1.06	0.62 - 1.82
Female	398	26 (6.5)		1.00	
**Age (844)**					
0 - 5 years	403	31 (7.8)	0.268	1.41	0.82 - 2.43
6 - 15 years	441	26 (5.8)		1.00	
**Family structure**					
**Number of adults (839)**					
1	42	5 (11.9)	0.187	2.02	0.76 - 5.36
> 1	797	50 (6.2)		1.00	
**Number of children (825)**					
1	349	24 (6.8)	0.777	1.10	0.63 - 1.91
> 1	476	30 (6.3)		1.00	
**Birth order (759)**					
Not the first	479	16 (6.8)	0.646	0.82	0.44 - 1.52
First	280	33 (5.7)		1.00	
**Mother's educational level (828)**					
No educated	13	3 (23.0)	**0.047**	4.49	1.20 - 16.84
Educated	815	51 (6.2)		1.00	
**Father's educational level (812)**					
No educated	14	6 (42.8)	**< 0.001**	12.26	4.08 - 36.82
Educated	798	46 (5.7)		1.00	
**Attendance of day care institution/Kindergarten (789)**					
Yes	640	43 (6.7)	0.592	0.82	0.42 - 1.60
No	149	12 (8.0)		1.00	
**Day care centre (< 3 years) (844)**					
Yes	193	14 (7.2)	0.745	1.11	0.59 - 2.07
No	651	43 (6.6)		1.00	
**Kindergarten (3-5 years) (844)**					
Yes	352	20 (5.6)	0.332	0.74	0.42 - 1.30
No	492	37 (7.5)		1.00	
***Helicobacter pylori *infection (821)**					
Yes	56	25 (44.6)	**0.037**	1.82	1.05 - 3.15
No	756	235 (30.7)		1.00	
**Chronic disease (837)**					
Yes	87	5 (5.7)	0.824	0.82	0.32 - 2.11
No	750	52 (6.9)		1.00	

* Numbers in parenthesis refers to sample size used for each variable after discharging children with missing data on the questionnaires. Statistically significant *P *values (< 0.05) are highlighted in boldface.

Children with pets were also more likely to be infected with *G. duodenalis *(*p *= 0.026; OR = 0.53; 95%CI: 0.31-0.93), with a borderline statistical association found for dogs (*p *= 0.050; OR = 0.50; 95%CI: 0.23-1.00) (Table 3). No association was found between giardiasis prevalence and some variables, such as gender, age, family structure (adults and children members in the family, children birth order), attendance of day care institution/kindergarten, children, medical status (chronic disease), housing characteristics (rural or urban, house/apartment, existence of current water, electricity, full bathroom, garden or terrace) (Tables 2 and 3).

**Table 3:** Prevalence of *Giardia duodenalis *infection according to housing characteristics.

Risk factor (total n*)	n	Positive results (%)	*p*	Risk Estimate (OR)	OR Confidence Interval (95%)
**Residence (833)**					
Rural	242	13 (5.3)	0.364	0.71	0.37 - 1.34
Urban	591	44 (7.4)		1.00	
**Housing (843)**					
Apartment	578	40 (6.9)	0.638	1.16	0.63 - 2.13
House	248	15 (6.0)		1.00	
**Water system (839)**					
Yes	834	55 (6.5)	0.293	0.28	0.03 - 2.57
No	5	1 (20.0)		1.00	
**Electricity (842)**					
Yes	840	55 (6.5)	0.129	0.07	0.00 - 1.14
No	2	1 (50.0)		1.00	
**System drainage (839)**					
Yes	834	54 (6.4)	**0.038**	0.10	0.02 - 0.64
No	5	2 (40.0)		1.00	
**Bathroom (841)**					
Yes	834	55 (6.5)	0.187	0.14	0.01 - 1.57
No	5	1 (33.3)		1.00	
**Garden (802)**					
Yes	176	11 (6.3)	0.740	0.84	0.43 - 1.66
No	626	46 (7.3)		1.00	
**Back yard (777)**					
Yes	201	8 (3.9)	0.073	0.49	0.23 - 1.06
No	576	45 (7.8)		1.00	
**Terrace (762)**					
Yes	206	13 (6.3)	0.750	0.87	0.46 - 1.66
No	556	40 (7.1)		1.00	
**Pets (839)**					
Yes	452	22 (4.8)	**0.026**	0.53	0.31 - 0.93
No	387	34 (8.7)		1.00	
**Dog (844)**					
Yes	246	10 (4.1)	0.050	0.50	0.23 - 1.00
No	598	47 (7.8)		1.00	
**Cat (844)**					
Yes	89	4 (4.5)	0.503	0.62	0.22 - 1.77
No	755	53 (7.0)		1.00	
**Rat (844)**					
Yes	29	1 (3.4)	0.715	0.48	0.07 - 3.62
No	815	56 (6.8)		1.00	
**Pets indoor**					
**Dog (844)**					
Yes	101	3 (3.0)	0.137	0.39	0.12 - 1.27
No	743	54 (7.2)		1.00	
**Cat (844)**					
Yes	56	2 (3.6)	0.577	0.49	0.12 - 2.08
No	788	55 (6.9)		1.00	
**Rat (844)**					
Yes	24	1 (4.2)	1.000	0.59	0.08 - 4.47
No	820	56 (6.8)		1.00	
**Pets outdoor**					
**Dog (844)**					
Yes	161	7 (4.3)	0.222	0.58	0.26 - 1.29
No	683	50 (7.3)		1.00	
**Cat (844)**					
Yes	37	2 (5.4)	1.000	0.78	0.18 - 3.33
No	807	55 (6.8)		1.00	
**Rat (844)**					
Yes	3	0 (0.0)	1.000	-	-
No	841	57 (6.7)			

* Numbers in parenthesis refers to sample size used for each variable after discharging children with missing data on the questionnaires. Statistically significant *P *values (< 0.05) are highlighted in boldface.

When a multiple logistic regression analysis was applied, using the five variables detected as significantly associated with *G. duodenalis *infection in the univariate analysis (p < 0.05), no additional support was found for this statistical significance (data not shown).

## Discussion

This study evaluated the prevalence of *G. duodenalis *infections in asymptomatic children in the District of Lisbon as well as potential risk factors for infection. Most epidemiological studies on *G. duodenalis *infection use microscopic detection, mainly due to its low cost, however, the low sensitivity of this test may result in the underestimation of the true prevalence of the parasite. In our study, we used both direct microscope detection and ELISA and the overall prevalence of *G. duodenalis *infection was 6.8%, with the ELISA presenting a much higher sensitivity, as expected [18-21]. Nevertheless, it should be stressed that the use of a single stool sample for diagnosis of this infection, as it was the case of our study, might underestimate the parasite prevalence, due to the intermittent excretion of cysts in stools.

Giardiasis is linked to the socioeconomic level of a country, with prevalence ranging between 2 and 7% in most industrialized regions and reaching 40% in developing countries [22]. Actually, in the American continent, with countries showing highly different levels of development, *G. duodenalis *prevalence ranges between 1.4% in the North and 24% in Latin America being the latest considered a high-risk area for the general population [23]. Recent studies in asymptomatic children reported *Giardia *prevalence ranging from 0.8% in Italy (0-14 years, direct microscope detection) [24], 1.3% in the UK (0-5 years, immunomagnetic and immunofluorescence microscopy) [25], 1.5% in Berlin (0-6 years, immunofluorescence microscopy) [26] to 31.9% in Russia (0-5 years, direct microscope detection) [27].

The prevalence of 6.8% found in our study, although not as high as the Russian value, is considerably higher than other European countries showing similar levels of welfare (i.e. Italy, UK, Germany *op. cit*.). On the other hand, considering the prevalence obtained using the direct exam only (1.9%), results are similar to the values reported in those countries. This discrepancy among methods may however, at least partly, be a consequence of the tendency of ELISA to overestimate positive results. Nevertheless, considering that Portugal reveals a prevalence of other gastrointestinal agents, such as *H. pylori*, much higher than that observed in other European countries, it is likely that *G. duodenalis *infection is higher as well [17].

In Portugal, Almeida *et al *[28] studying a group of 177 asymptomatic children from the north region of Portugal reported a prevalence of 4% using the direct examination with sample concentration. Considering that socioeconomic conditions are relatively similar in both regions, the differences found in both studies, also reinforces the idea that results are influenced by the different accuracy of ELISA when compared with direct methods. The prevalence of intestinal parasites is a direct consequence of a set of constant factors, particularly climate, food and water supplies, personal and community hygiene, sanitation, proximity to both domestic and wild animals, and socioeconomic condition, all play a role regarding the risk of exposure to intestinal parasites [23]. The present study showed similar results regarding the factors enhancing the risk of giardiasis, revealing that the higher risk for *G. duodenalis *infection was related to the lower parents' educational level. Results match previous studies [29-31], which indicated that maternal educational level was inversely correlated with the risk of children infection. Similar studies in Uganda showed that the mother's educational level was the best predictor of health and nutrition inequalities among children in rural communities [31]. Furthermore, Nematian *et al *[29] highlighted that the higher the educational level of the mothers, the lower the parasitic infection rate in children in Iran. Interestingly our study revealed that an illiterate father represented a risk for *G. duodenalis *infection almost three times higher when compared with an illiterate mother. This result may suggest that father's educational level reflects more clearly the family socio-economic level, corroborating the known association of *Giardia *infection with a low social status and consequently poorer sanitary conditions.

Another association found was between *H. pylori *infection and *Giardia *infection, which may indicate that they both share the same route of transmission. Actually, the fecal-oral transmission route has been proposed for the two microorganisms [32] and the prevalence of *H. pylori *in Portuguese children has been reported to reach 50% [17,33]. The co-infection with these two agents has been previously reported among Brazilian healthy children, in a population with high levels of *H. pylori *infection [32]. Other authors showed the existence of *H. pylori *infection in 37 of 41 (90.2%) patients with gastric giardiasis [34], suggesting that this condition increases the susceptibility to *H. pylori *infection. Other authors sustain that *H. pylori*-induced chronic gastritis may increase the susceptibility to *G. duodenalis *infection [32]. Present results showed that 8 in 11 children acquired *H. pylori *infection after giardiasis, supporting the first hypothesis. On the other hand, the reciprocal antagonism of T-helper-1 (Th-1) and Th-2 type immune responses suggests that parasitic infection may ameliorate disease where a Th-1 type response dominates, such as *H. pylori*-induced gastritis and gastric carcinoma [35]. Thus, the association between *G. duodenalis *and *H. pylori *and its benefits or injuries is a promising line of research.

The zoonotic transmission of *G. duodenalis *cysts from animals to humans has been raised, although many questions still remain. Among animals, dogs are the most studied species for giardiasis. Evidence of zoonotic transmission among humans and dogs has been reported by Traub *et al *[36] based on epidemiological data combined with molecular techniques. In many countries the role of dogs as a definitive *G. duodenalis *host has been widely recognized as a public health problem. The present study also shows a positive association regarding the prevalence of this parasite and the contact with pets, especially dogs. Nevertheless, the multiple logistic regression did not confirm the univariate significant associations, likely due to the fact that a reduced number of individuals for which an association was found, thus hindering the disclosure of significant associations. Moreover, the likelihood that the results are the outcome of interactions and confounding effects that are not evident in a simple comparison of two treatment groups may also explain the absence of significant associations.

The literature shows no agreement on paediatric age and gender as associated factors. Nevertheless, some studies referred to a positive association [29,37,38] with female gender in adulthood, mostly due to the nursing activity in communities with a high infection prevalence among children [38]. On the other hand several authors considered age an important risk factor for this infection, finding higher prevalence in young adults and children [16,29,38-44]. The present study showed no association either for gender or age regarding the prevalence of *Giardia *infection. Longitudinal and case-control studies carried out in Israel, Brazil and Kenya reported a higher risk of *G. duodenalis *infection for children after the first year of life [16]. The reasons for this age-associated risk are not well understood, although the lack of acquired immunity, more adventurous travel styles, or different diet regimes have been implicated [31,40]. Although our study did not reveal a significant association with *Giardia *prevalence and age, the prevalence was slightly higher in younger children (0 - 5 years).

## Conclusion

The present study revealed the high prevalence of *G. duodenalis *infection in asymptomatic children residing in the District of Lisbon. The factor more directly associated with the risk of giardiasis infection was shown to be the parents' educational level, suggesting that an increment in parents' education is likely to have a positive influence on the well being of Portuguese children. Co-infection with *G. duodenalis *and *H. pylori *was detected and seems an important issue deserving further investigation.

Studies regarding giardiasis prevalence show worldwide, a clear gap from developed to developing countries, mainly reflecting socioeconomic level. Nevertheless, this approach is somehow limited as even in developed countries the trilogy of socioeconomic-educational level/sanitation/veterinary care with pets, was shown to be an association to follow in order to prevent giardiasis.

## Competing interests

The authors declare that they have no competing interests.

## Authors' contributions

MO and LM conceived the study and collected all samples. CJ, AV, IF, SG performed all the experiments, analyses and wrote the paper with RT and HA. BN was involved in the statistical analysis. All authors read and approved the final manuscript.

## Acknowledgements

The authors are grateful to Assunção António for laboratory assistance. We like to thank Professor José Guerreiro for the manuscript revision. Very special thanks to all the dedicated staff of the health care centres and to all the children and their parents for participating in this study.
